# The efficiency and use of a reciprocating system aid for standing and walking in children affected by severe cerebral palsy

**DOI:** 10.3389/fped.2024.1447512

**Published:** 2024-12-05

**Authors:** Priscilla Avaltroni, Yury Ivanenko, Carla Assenza, Hilenia Catania, Michele Coluccini, Giovanni Morone, Daniela Morelli, Germana Cappellini

**Affiliations:** ^1^ Laboratory of Neuromotor Physiology, Istituto di Ricovero e Cura a Carattere Scientifico Fondazione Santa Lucia, Rome, Italy; ^2^ Department of Systems Medicine and Center of Space Biomedicine, University of Rome Tor Vergata, Rome, Italy; ^3^ Department of Pediatric Neurorehabilitation, Istituto di Ricovero e Cura a Carattere Scientifico Fondazione Santa Lucia, Rome, Italy; ^4^ Department of Developmental Neuroscience, Istituto di Ricovero e Cura a Carattere Scientifico Fondazione Stella Maris, Pisa, Italy; ^5^ Department of Life, Health and Environmental Sciences, University of L'Aquila, L'Aquila, Italy

**Keywords:** locomotion, children with cerebral palsy, robotic assistive devices, exoskeleton, rehabilitation

## Abstract

Cerebral Palsy (CP) is a leading cause of childhood motor disability, making independent walking a crucial therapeutic goal. Robotic assistive devices offer potential to enhance mobility, promoting community engagement and quality of life. This is an observational report of 22 cases of children with CP in which we evaluated the Moonwalker exoskeleton (a dynamic moving aid system) usability, functional changes, and caregivers' perspectives based on the International Classification of Functioning (ICF). All children (aged 2–8 years, with a severe gait impairment and inability to use a conventional walker) underwent Moonwalker training for 20 sessions, followed by home use for five months. Post-treatment, majority of children showed improved endurance assessed by the 10-m walk test with a notable involvement of the upper trunk and arm movements for gait assistance. Many of them achieved rather remarkable results reaching a velocity of ≥0.5 m/s given the constraints of the walking exoskeleton and the children's size, while at admission all children walked at a speed of less than 0.5 m/s. Several positive environmental factors and family adherence were noted, as assessed by ICF in a subgroup of children. This study on a sample of children demonstrated that the Moonwalker exoskeleton allows walking and training at home in children with severe CP, enhancing development, social interaction, and endurance, while being well-received by families.

## Introduction

Cerebral palsy (CP) is the most common form of motor disability in childhood, marked by non-progressive movement and posture disorders resulting from prenatal or neonatal brain injury (1). It is often characterized by muscle weakness, impaired coordination muscle and spasticity accompanied by hypertonia, hyperreflexia, clonus, spasms and co-contraction (2). The motor disorders of CP are often accompanied by cognitive deficits, disturbances of sensation, perception, communication, and behavior, as well as epilepsy, and secondary musculoskeletal problems (3, 4). The overall prevalence of CP in Europe is approximately 2 subjects out of 1,000 (5) and 70% of children with CP experience walking problems (from minimal disability to the need for assistive walking devices) (6).

The development of efficient and independent walking is an important therapeutic goal for children with CP (7–10) and plays a fundamental role in social development (11) and psychological function (12). Children with CP are often less mobile and interactive than their peers (13). This lack of mobility can have a negative impact on overall development, social interaction and social status (14, 15). Moreover, children with CP who are able to walk demonstrate low walking ability, energy efficiency and difficulties in adaptation (16–18), which require coordination adjustments, visual guidance, enhanced balance and anticipatory control, necessitating significant cortical contribution (19). Low walking abilities affect social interactions and community-based activities (20).

Advances in biotechnology could play a central role in developing various devices and services for children with CP to maximize physical capabilities, maintain functional skills into adulthood, and enhance walking. For example, locomotor training with partial body weight support on a treadmill may be considered as therapeutic tools for gait improvement in children with CP (21–25). In addition, combining gait training with spinal cord neuromodulation may also improve locomotor functionality (26, 27). Positive effects of repetitive locomotor exercise on gait characteristics and walking ability in CP (8, 28) have been also obtained with the use of a wearable exoskeleton (29). Early stimulation or training of the locomotor function holds the potential to modify the trajectory of motor development, enabling infants with CP to engage in motor learning experiences characterized by playful self-discovery and exploration, typical for healthy children (19).

Robotic gait training represents a new frontier in the world of pediatric rehabilitation (30). There are interesting recent technological assistive solutions for implementing early locomotor therapy in children with CP younger than 2 years of age, such as a recently developed powered exoskeleton “ExoAtlet” helping young children (∼2 years) to learn how to walk (19), and a pediatric skateboard (31) or self-initiated prone progression crawler robotic system (32) for infants. According to the idea behind the latter devices, early quadrupedal training may enhance interventions intended to accelerate the onset of independent walking in infants with CP and developmental delays. Also, given our understanding of neuroplasticity and critical developmental windows, training in the early sensitive period for maturation (<2 years) may help to optimize infant motor and cognitive plasticity and enhance more effectively their locomotor function (19, 33–35). For older children, various exoskeletons (powered and passive models with differing levels of movement freedom) are being developed to address specific motor and support needs in pediatric rehabilitation. Some have rather distinct modes of operation, like the Lokomat (36) or Gait Trainer (8), which are actually stationary devices that employ respectively, a treadmill for walking or foot plates controlling feet endpoint trajectories, while other robotic systems for overground walking merely help local joints or have limitations on motion and balance [e.g., (29, 30, 37)]. Furthermore, many studies utilizing assisted gait training measured gait outcome parameters pre- and post-intervention and not the direct effect of the exoskeleton on gait. Compared to tethered robots, overground powered exoskeletons, such as Angel Legs M20 (38), ATLAS2030 (39, 40), ExoAtlet Bambini (41), appear to be more effective for dynamic balancing and navigating in a more natural setting, although they are more pricey, or made for older children, or difficult to prescribe for use at home due to availability or safety concerns. Some other exoskeletons are integrated with a wheeled walker frame, such as the Trexo Plus (42) or CPWalker (43), and use active motors to facilitate limb movements. In contrast, passive, non-actuated exoskeletons, such as the David Hart Walker (44), Norsk Funktion-walker (45, 46), Moonwalker exoskeleton (https://progettiamoautonomia.it/prodotto/moonwalker-reciprocating-system/), are more accessible, less costly, and reasonably easy to use for extended periods of time in a home setting. In addition, in contrast to conventional walkers, the latter devices, that combine walking and standing aid, were designed to allow children with severe cerebral palsy to maintain an upright posture, ambulate with hands-free support, providing postural stability and increasing the capacity for activity and participation, but above all for independent walking. Also, the reciprocating gait mechanism supports coordinated left-right movements, promoting a more physiological walking cycle. In this study, we used the Moonwalker exoskeleton: its features, combined with adjustability to accommodate growth, make it suitable for a long-term use and active engagement in the training process.

Before prescribing a robotic device, we should consider environmental aspects like cost, accessibility, the device's ability to adjust to the patient's changing physical size, and the device's social acceptance. It's important to deeper understand the impact of these assistive devices also on personal, environmental contextual factors and on multiple components of health and functioning as defined by the World Health Organization's Classification of Functioning, Disability and Health (ICF) (47). A recent study by Paleg et al. (48) highlights the dynamic interaction between the F-words (functioning, family, fitness, fun, friends, and future) for childhood development included in the ICF model and the use of standing and stepping devices. ICF is a classification system, which aims to provide a scientific basis for understanding and studying health and health-related states, outcomes, determinants, and changes in health status and functioning in order to improve communication between different users, including people with disabilities. It defines health as a result of a dynamic interaction between an individual's domains (Body structure and Function, Activity and Participation) and two contextual factors (Environmental and Personal). The criteria of the ICF, even more so in the version specific to children and young adults (ICF-CY), can meet the needs of the patient and his or her family who find themselves struggling with participating in paediatric neurorehabilitation.

The purpose of the current study was to examine the feasibility and efficiency of the Moonwalker exoskeleton that combines walking and standing aid in children with severe CP, that are unable to use a conventional walker, both in clinical setting and at home. In line with some previous studies that used walker orthoses (44, 45), here we further evaluated the use and effectiveness of such device and added other parameters for its evaluation. In particular, we evaluated the child's functional changes after they utilized it, in a clinical environment and at home, with the 10-m walk test, and focusing on the achieved walking speed as an important indicator of efficient interaction with environment and mobility. In a subset of participants, we also assessed the Level of Sitting Scale (before and after training) and explored the perspectives of family members and professional caregivers on relevant areas of impairment and functional abilities, as operationalized by the ICF CY.

## Materials and methods

### Participants

A series of twenty-two cases was included in this study. All 22 children with a clinical diagnosis of congenital CP (age range 2–8 years old) were recruited from the Department of Paediatric Neurorehabilitation of IRCCS Santa Lucia Foundation. CP diagnosis was confirmed according to medical history, brain magnetic resonance results, and clinical examination. Participants characteristics are detailed in Table 1. This project was approved by the IRCSS Santa Lucia Foundation Ethics Committee (protocol CE/PROG.430). All of the children who were admitted to this study had been receiving conventional therapy for a few months or years prior, and their clinical scores were pretty stable when they started the study. Throughout the study, every child in the sample kept up their current rehabilitation treatments. Each child's parents were informed about the study's purpose, duration and structure, and informed written consent was obtained from the parents of all children. Inclusion criteria were: spastic quadriplegic CP diagnosis, a severe gait impairment (most of our children showed the Gross Motor Function Classification System – GMFCS – Level IV and V, Table 1) and inability or difficulty to use a conventional walker. The device was proposed to children not having ambulatory abilities independently or with conventional walking aids, due to their clinical condition (lack of or partial control of head and trunk, poor supporting reaction, spasticity, insufficient upper extremity control to stand and walk with a traditional walker/rollator, reduced capacity of autonomous progression). Exclusion criteria were: hip dislocation, flexion contractures of the hip, pain on weight-bearing, lower limb deformities that cannot be corrected with orthotics, severe scoliosis, and knee >20° and sever cognitive deficit corresponding to an intelligence quotient (I.Q.) less than 49. Ankle plantarflexor muscle spasticity was evaluated by the Modified Ashworth Scale (MAS). The assessments of GMFCS, MACS (Manual Ability Classification System), CFCS (Communication Function Classification System) and MAS (Table 1) were carried out by experienced physiotherapists in accordance with the manuals available for these instruments.

**Table 1:** Characteristics of children.

	Gender	Age at admission, years	Lesion characteristics						GMFC-s	MACS	CFCS	MAS (R/L)
			Lesion type			Lesion site						
			PWM	CDGM	M	ANT	POST	not def.				
Child 1	M	2.1	1	0	0	0	0	1	IV	III	III	3/3
Child 2	F	2.6	0	0	1	0	0	1	IV	II	IV	3/2
Child 3	F	2.6	1	0	0	1	0	0	V	III	IV	2/2
Child 4	M	2.6	0	1	0	1	1	0	V	V	V	2/2
Child 5	F	2.6	0	0	1	0	1	0	V	IV	IV	2/2
Child 6	F	2.8	1	0	0	0	1	0	IV	III	IV	4/3
Child 7	M	3	1	0	0	0	0	1	IV	I	I	3/3
Child 8	M	3.5	–	–	–	–	–	–	V	V	IV	4/4
Child 9	M	3.5	1	1	0	1	1	0	IV	IV	III	2/2
Child 10	M	3.5	1	0	0	0	1	0	V	IV	V	3/3
Child 11	M	3.8	1	0	0	0	0	1	IV	III	I	3/2
Child 12	M	4.1	1	0	0	0	1	0	IV	III	IV	3/3
Child 13	M	4.6	0	1	0	1	1	0	IV	IV	V	1/1
Child 14	F	4.6	1	0	0	0	0	1	IV	III	II	3/3
Child 15	M	5.9	–	–	–	–	–	–	II	I	I	3/3
Child 16	F	6.1	–	–	–	–	–	–	V	V	IV	3/3
Child 17	M	6.3	1	0	0	0	1	0	IV	V	IV	4/4
Child 18	F	6.9	0	0	1	0	0	1	IV	IV	IV	1/1
Child 19	M	7	0	0	1	0	1	0	V	IV	IV	3/4
Child 20	F	7.2	1	0	0	0	0	1	III	II	II	1/1
Child 21	M	7.5	1	0	0	0	0	1	IV	II	I	3/4
Child 22	M	8.4	1	0	0	0	0	1	IV	I	I	3/3

PWM, periventricular white matter lesions; CDGM, cortical and deep grey matter lesions; M, miscellaneous (white and grey matter lesions); ANT, anterior lesions; POST, posterior lesions; not def, not defined; GMFC-s, gross motor function classification system; MACS, manual ability classification system; CFCS, communication function classification system; MAS, modified Ashworth scale (the rater graded each ankle plantarflexion spasticity).

### The dynamic moving aid system (“moonwalker” exoskeleton)

**Figure 1:**
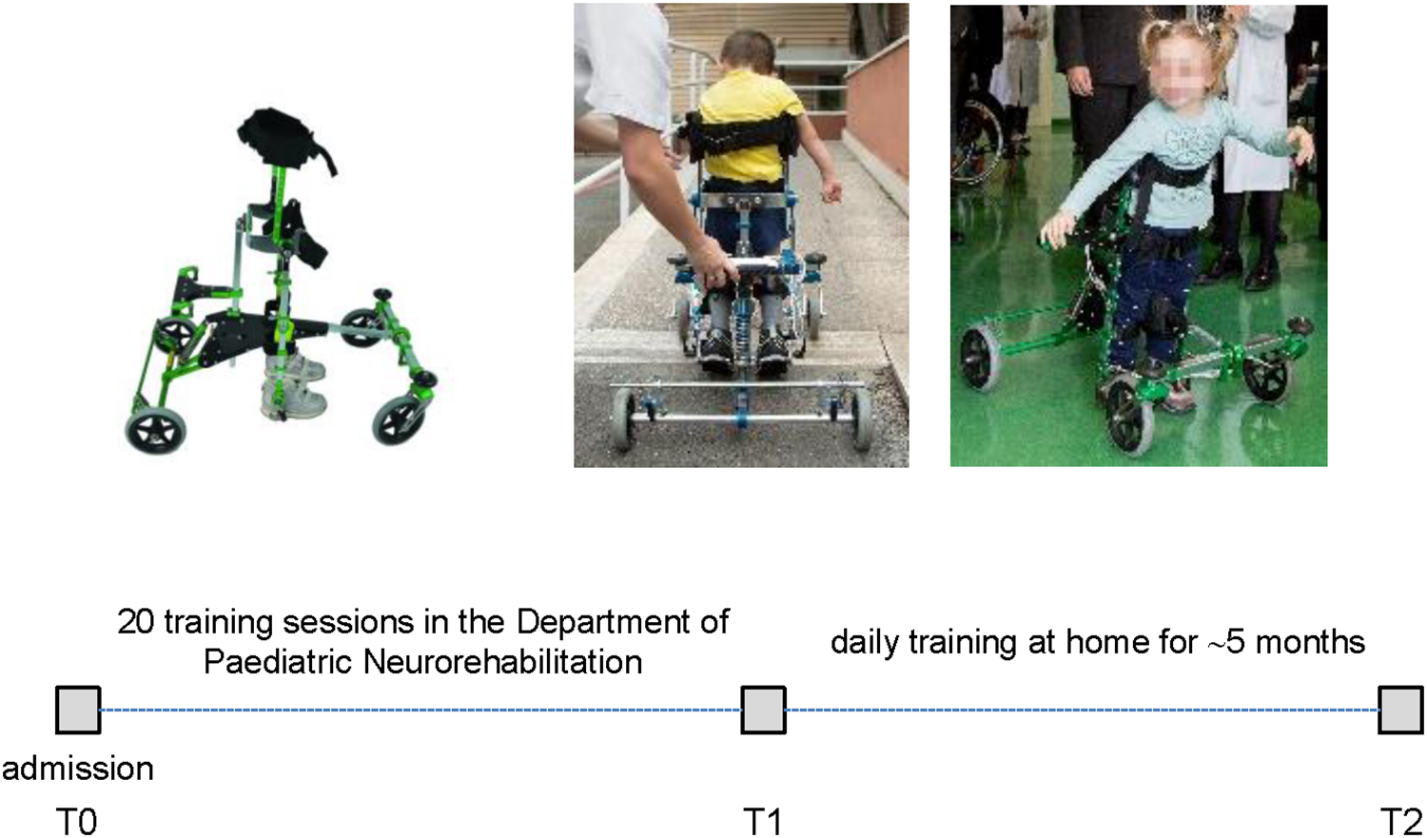
Schematic timeline and overview of performed sessions of training and evaluation of the moonwalker exoskeleton. T0—first use of Moonwalker in the Department of Pediatric Neurorehabilitation (admission). T1—termination of 20 sessions of training in the rehabilitation department. T2—the time of the completion of at-home activities and prescription after 5 months of usage at home.

The Moonwalker exoskeleton is a reciprocating aid system supporting upright standing and locomotion under gravitational load (https://progettiamoautonomia.it/prodotto/moonwalker-reciprocating-system/) (Figure 1, upper panels). This aid promotes the development of functional residual resources necessary for the walking cycle in children with neuromotor disabilities. Moonwalker is composed by two functional units: (1) the orthotic part with a trunk-pelvis-knee-ankle-foot connected to (2) a walker with four wheels. The orthotic part (shank, thigh and trunk support segments, as well as the base of support—the distance between the wheels) is adjustable for the length of the child's leg and body size. Additionally, spring mechanisms, which are adjustable and attached to the knee joints to deliver a horizontal force to each limb, regulate propulsion thrust and leg movements in alternating left-right coordination manner according to individual walking characteristics, ensuring stability, safety, and optimal body alignment. The device is designed for children between the heights of 80 and 140 cm and weighs ∼11.5 kg (excluding accessories), with a maximum subject weight capacity of 30 kg. Depending on the axle layout, the distance between the frontal and rear (12.5–17.5 cm in diameter) wheels might vary from 57 to 87 cm. The exoskeleton is designed to be relatively simple to put on and take off; a parent or a trained therapist can do it in ∼5 min.

The main innovation of Moonwalker is the ability to maintain lower limb load-bearing and enabling hands-free movements, representing a significant advancement in assistive technology for children with CP with severe gait impairments. By promoting unrestricted movement of the upper limbs, the Moonwalker enhances mobility and engagement in daily activities, provides assistance to the lower extremity joints making muscles flexion-extension easier despite a small residual lower limb function, as well as support and postural stability, thereby fostering greater independence and social participation (48).

### Protocol

Figure 1 schematically illustrates the overview of performed sessions of training and evaluation of the Moonwalker exoskeleton. The study was proposed in addition to a conventional rehabilitative individualized treatment (twice a week for an hour each). In the Department of Paediatric Neurorehabilitation, the children who were recruited had twenty sessions of walking exercises using the Moonwalker, followed by thirty minutes of traditional motor therapy. The training then continued at home for about 5 months. We assessed outcome measures (see below) at the beginning (T0), following 20 training sessions (T1), and following the completion of at-home activities (T2) (Figure 1).

### Outcome measures

We assessed mobility using the Moonwalker with the 10 Meter Walk Test (10 MWT) (49) at T0, T1 and T2. In each trial, an experimenter verbally provided a start signal for forward walking. Participants were encouraged to walk as quickly as possible along a 10-m hallway while time was recorded. If a child was unable to complete 10 m in one minute, the distance travelled was reported (to avoid long trials). Thus, if the child walked the 10 m distance in less than 1 min, the actual trial duration was recorded; if he/she did not complete the 10 m distance in 1 min, the actual distance was used. The distance was measured with a measuring tape and the duration with a stop watch. Based on the results derived from the mobility assessment, we specifically focused on the maximally attained walking speed. In each trial, the walking speed was computed as the total travelled distance divided by the time spent travelling. The 10WT was repeated several times (typically 5–7 times) and we reported the best performance across these trials.

Additionally, in a subset of children (*n* = 7) the Level of Sitting Scale (LSS), which is a component of the Seated Postural Control Measure and acts as a general indicator of the child's sitting abilities (50, 51), was used to evaluate the sitting position at T0 and T2. The LSS levels are determined by the amount of support required to maintain a sitting position and, for those children who can sit independently without support, the stability of their sitting posture.

In a subset of children (*n* = 7) after usage at home (T2) with consistent parental involvement and availability for follow-up assessments, we also gathered the parents’ satisfaction using the Quebec User Evaluation of Satisfaction with Assistive Technology (QUEST) questionnaire, an index designed to measure the level of satisfaction attribute to assistive technologies. It focused on various characteristics of the device such as, dimensions (item 1), weight (item 2), ease of adjustment (item 3), safety and security (item 4), durability (item 5), ease of use (item 6), comfort (item 7), effectiveness (item 8), and on services as service delivery (item 9), maintenance (item 10), information-attention (item 11) and continuing support services (item 12) (52). For each case, the rehabilitation team, in consultation with the child's family, evaluated both the functional ability of the child to use the Moonwalker and the environmental factors affecting its use. The selection of environmental factors was guided by considerations of physical and social conditions that could either enable or limit the use of the device at home, such as available space for safe movement, family support, and access to assistance. The decision to prescribe or not prescribe the device was made collaboratively in team meetings, where consensus was reached based on the child's individual needs and the environmental factors discussed with the family. In particular, the parents reported the mean amount of time the Moonwalker was used per day. The evaluation outcome included also the application of the International Classification of Functioning, Disability and Health (ICF-CY) scale. ICF is a classification of health-related domains (53). As the functioning and disability of an individual occurs in a context, ICF also includes a list of environmental factors (E150, Design, construction and building products and technology of buildings for public use; E155, Design, construction and building products and technology of buildings for private use; E310, Immediate family; E340, Personal care providers and personal assistants; E355, Health professionals; E410, Individual attitudes of immediate family members; E525, Housing services, systems and policies; E580, Health services, systems and policies) (https://www.who.int/standards/classifications/international-classification-of-functioning-disability-and-health). This classification system was important to understand prescriptive adequacy. If the child's functioning profile was negatively influenced by environmental factors, which conditioned the training, the device was not prescribed.

The current study was aimed to investigate the potential long-term use and benefits of the Moonwalker exoskeleton for children with severe cerebral palsy and inability to use a conventional walker, as well as the mobility and performance results. Given the heterogeneity of a sample of children, we mostly show individual and averaged scores and walking performance for all participants, along with the ICF and QUEST-based Moonwalker usage evaluation.

## Results

### General performance

**Figure 2:**
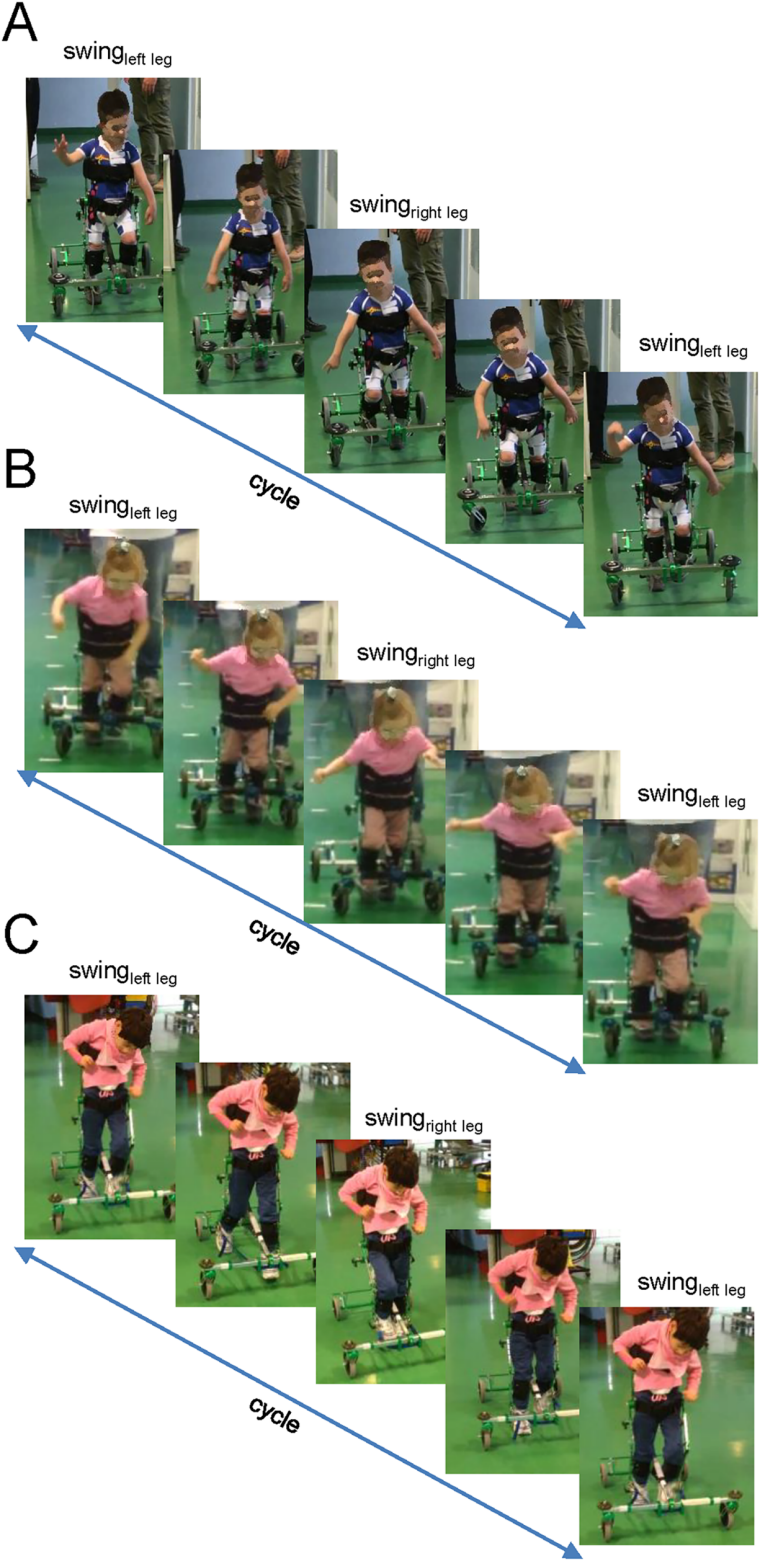
Examples of gait cycles during walking in the moonwalker exoskeleton in three children. The illustration consists of five frames evenly spaced along a single cycle. Note how the children in panels (**A**,**B)** (child 6 and 7, respectively, Table 1) significantly involve their upper trunks and arms for assistance, whereas the child in panel **(C)** (child 5) does not.

The Moonwalker was used by twenty-two participants. Figure 1 (upper panels) illustrates the examples of walking in the Moonwalker exoskeleton in children with CP. Given the design of the exoskeleton, which offered stability through trunk support in addition to a wide base of support (4 wheels), children never fell and were free to use their arms to accompany leg movements or interact with the environment. In particular, the majority of children (about 80%, assessed by videorecordings made during some trials) exhibited a strategy that engaged upper trunk rotations or lateral movements to assist leg motion. These movements often involved synchronous motions of the head and arms in a rhythmic manner to facilitate progression in walking (Figure 2A,B). Other children (typically with poor trunk balance control) did not engage prominent trunk/head/arm rotations (Figure 2C) or used small trunk tilts in the sagittal plane to assist gait (although we did not quantify it). Nevertheless, these observations (Figure 2) demonstrated how children with severe cerebral palsy may use the Moonwalker exoskeleton in a variety of ways, each of which reflects their individual abilities and adaptations to the robotic device. Generally, children succeeded to complete the training protocol (Figure 1, lower panel). Only in one case (case 10) the training with the device stopped during the use at home due to negative environmental factors (E150, E155, E410, E525, see Table 3). Twenty-one other children made regular use of the exoskeleton both in the rehabilitation centre and at home.

### Evaluation of functional changes using 10 m walk test before and after training

Table 2 shows the results of testing the ability to move while using the exoskeleton (10MWT). In terms of walking abilities, at the first use of the Moonwalker exoskeleton in the Department of Paediatric Neurorehabilitation (T0), nine children could not succeed in walking ten meters: four of them were unable to walk at all and the other five could only manage 3–9 m in a minute. After 20 sessions of training in the Department of Paediatric Neurorehabilitation (T1), seventeen children were able to walk ten meters in less than 1 min, on average for 30 s. When testing at T2, one participant (child 10) failed to complete treatment due to the lack of motivation, and two children failed to show walking. Nevertheless, most children at the time of prescription after five months of training at home (T2) improved endurance by traveling ten meters in less time or by increasing the distance walked in one minute with respect to T0.

**Table 2:** Performance of the 10 m walk test [L–distance, T–duration, V–walking speed (L/T)] with the moonwalker exoskeleton and evaluation of sitting abilities using the LSS before and after training. Some subjects walked the 10 m distance in less than 1 min (actual trial duration is indicated in parentheses), while others failed to complete the 10 m distance for 1 min (actual distance is indicated).

	10MWT						LSS	
	T0		T1		T2		T0	T2
	L (T)	V, m/s	L (T)	V, m/s	L (T)	V, m/s		
Child 1	3 m (60 s)	0.05	7 m (60 s)	0.11	9 m (60 s)	0.15	3	3
Child 2	10 m (50 s)	0.20	10 m (50 s)	0.20	10 m (23 s)	0.43	–	–
Child 3	0 m (60 s)	0	3 m (60 s)	0.05	0 m (60 s)	0	–	–
Child 4	10 m (50 s)	0.20	9 m (60 s)	0.15	8 m (60 s)	0.13	–	–
Child 5	10 m (50 s)	0.20	10 m (20 s)	0.50	10 m (18 s)	0.55	–	–
Child 6	6 m (60 s)	0.10	10 m (37 s)	0.27	10 m (17 s)	0.59	4	4
Child 7	6 m (60 s)	0.10	10 m (50 s)	0.20	10 m (18 s)	0.54	–	–
Child 8	10 m (30 s)	0.33	10 m (25 s)	0.40	10 m (20 s)	0.50	–	–
Child 9	10 m (25 s)	0.40	10 m (17 s)	0.59	10 m (15 s)	0.67	3	4
Child 10	0 m (60 s)	0	6 m (60 s)	0.10	–	–	2	2
Child 11	10 m (23 s)	0.43	10 m (16 s)	0.62	10 m (12.5 s)	0.80	–	–
Child 12	0 m (60 s)	0	0 m (60 s)	0	0 m (60 s)	0	3	4
Child 13	6 m (60 s)	0.10	10 m (43 s)	0.23	8 m (60 s)	0.13	3	3
Child 14	10 m (20 s)	0.50	10 m (22 s)	0.45	10 m (20 s)	0.50	–	–
Child 15	0 m (60 s)	0	10 m (28 s)	0.35	10 m (21 s)	0.47	–	–
Child 16	9 m (60 s)	0.15	10 m (38 s)	0.26	10 m (25 s)	0.40	–	–
Child 17	10 m (60 s)	0.17	10 m (30 s)	0.33	10 m (25 s)	0.40	2	3
Child 18	10 m (40 s)	0.25	10 m (22 s)	0.45	10 m (20 s)	0.50	–	–
Child 19	10 m (38 s)	0.26	10 m (20 s)	0.50	10 m (18 s)	0.55	–	–
Child 20	10 m (25 s)	0.40	10 m (20 s)	0.50	10 m (18 s)	0.55	–	–
Child 21	10 m (40 s)	0.25	10 m (39 s)	0.25	10 m (15 s)	0.66	–	–
Child 22	10 m (30 s)	0.33	10 m (25 s)	0.40	10 m (14 s)	0.71	–	–

LSS, level of sitting scale: Level 0, unpeaceable; Level 1, supported from head downward; Level 2, supported from shoulders or trunk downwards; Level 3, supported at pelvis; Level 4, maintains position, does not move; Level 5, shifts trunk forward, re-erects; Level 7, shifts trunk laterally, re-erects.

**Figure 3:**
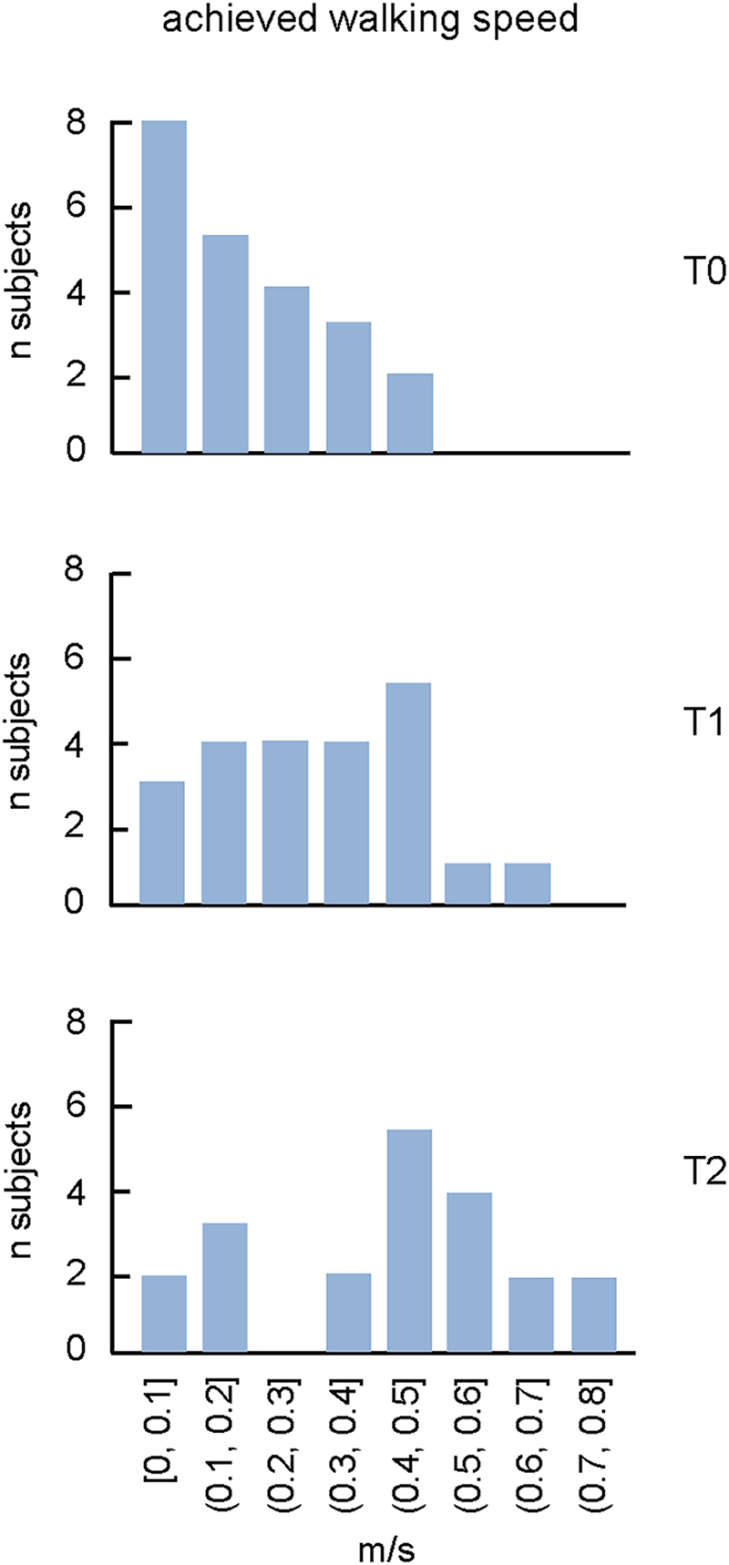
The proportion of subjects with achieved walking speed during walking in the moonwalker exoskeleton at **T0**, **T1** and **T2**. Note an increase of the maximally achieved speed and the corresponding number of subjects with training.

In terms of walking speed achieved with the exoskeleton, there were notable improvements. Remarkably, sixteen children attained a velocity of 0.4–0.8 m/s at T2 (Table 2), which is likely near optimal considering the limitations of the passive walking exoskeleton and the children's size. With trunk support and stability for the lower extremity joints, such speeds allow for functional movement and involvement in daily activities, which promotes increased independence and social interaction, as well as motivating children to further use the Moonwalker exoskeleton at home (see the next section). There were significant gains in the exoskeleton-assisted walking speed both following training in the hospital (at T1) and after training at home (at T2). We did not find significant correlations between the achieved walking speed at T2 and the child' age (*r* = 0.38, *p* = 0.09) or ankle plantarflexor muscle spasticity [*r* = 0.26, *p* = 0.25, spasticity of the right and left legs (see MAS in Table 1) was averaged for performing this correlation], likely due to multiple factors affecting walking inability. Nevertheless, the walking speed reached (∼0.5–0.7 m/s) is likely functional for Moonwalker exoskeleton-assisted locomotion in the age group investigated. As shown in Figure 3, at T0, more than half of the children could hardly if all move (at speeds ranging from 0 to 0.2 m/s), whereas at T2, more than half of the children achieved speeds ranging from 0.4 to 0.8 m/s.

In a subgroup of participants (*n* = 7), we evaluated the sitting capability (LSS) before (T0) and after treatment (T2). In terms of sitting skills, at T0, two children displayed LSS levels 2, four children displayed levels 3, and one child displayed level 4. At T2, while sitting performance was still poor in all children (levels 2–4), nevertheless, three children demonstrated an improvement in the trunk control (Table 2). Improvements in the trunk control (LSS) did not necessarily follow improvements in the 10MWT (Table 2).

### ICF and QUEST assessments following training

At the end of the performed training sessions, in a subset of children we also evaluated the Moonwalker functionality and satisfaction by medical staff and parents by means of the ICF and QUEST (Table 3). Taking into account the child's profile and at-home use, the medical staff recommended a prescription to continue using it, influenced by the absence of negative environmental factors and the presence of positive environmental factors, as well as adherence to treatment and parental involvement. Three children presented limitations in the use of the device: e.g., concerning the design, construction and building products and technology of buildings for private use (small house with 3 levels - E155), individual attitudes of immediate family members (poor investment - E410), or health services (E580). As a result, two of them were not prescribed for further usage of the device. One of them (child 1) had a negative environmental factor (E155), but the family had a positive and proactive attitude (E410) seen by inserting the device in other environments (for example at school - E150) and was prescribed. All participants had positive environmental factors interaction with immediate family (E310), and health professionals (E355). Furthermore, participants had positive environmental factors with personal care providers and personal assistants (E340 - 4 children), the design, construction and building products, and technology of buildings for public (E150 - 1 child), and private use (E155 - 1 child), as well as housing services, systems and policies (E410 - 1 child). Four children showed the absence of negative environmental factors, the presence of positive environmental factors and good adherence of the family to the rehabilitation program. Based on the above results, five children were prescribed to use the Moonwalker exoskeleton. Parents often expressed high levels of satisfaction and functionality with the Moonwalker. On average, the parents’ satisfaction (QUEST) regarding the assistive device was 3.9, and regarding the services provided - 4.8 (Table 3).

**Table 3:** Parents’ satisfaction by QUEST and prescription based on the use at home and ICF children profile at T2.

	Parents’ satisfaction by QUEST														Use time per day at home (minute)	Positive environmental factors	Negative environmental factors	Prescription for future use
	Device evaluation									Service evaluation								
	Item 1	Item 2	Item 3	Item 4	Item 5	Item 6	Item 7	Item 8	Mean	Item 9	Item 10	Item 11	Item 12	Mean				
Child 1	2	4	2	5	5	4	4	4	3.8	5	5	5	5	5	19	E150, E310, E355, E410	E155	yes
Child 6	5	4	3	5	5	3	5	5	4.4	5	5	5	5	5	60	E310, E340, E355		yes
Child 9	3	4	2	2	3	2	5	5	3.3	5	4	5	5	4.8	60	E310, E340, E355		yes
Child 10															Drop out	E310, E355	E150, E155, E410, E525	no
Child 12	3	4	3	5	4	4	3	5	3.9	5	5	5	5	5	n.a.	E310, E340, E355	E580	no
Child 13	5	5	5	3	3	5	5	5	4.5	5	5	5	5	5	30	E310, E355, E510		yes
Child 17	5	5	2	2	1	4	4	5	3.5	4	4	4	4	4	40	E310, E340, E155, E355		yes
Mean									3.9					4.8				

QUEST, Quebec User Evaluation of Satisfaction with Assistive Technology; 1, not satisfied at all; 2, not very satisfied; 3, more or less satisfied; 4, quite satisfied; 5, very satisfied; n.a., not available (the parents did not record it). Items 1–8 refer to the device evaluation, while items 9–12 refer to the service evaluation (52). ICF, international classification of functioning, disability and health (https://www.who.int/standards/classifications/international-classification-of-functioning-disability-and-health): E150, design, construction and building products and technology of buildings for public use; E155, design, construction and building products and technology of buildings for private use; E310, immediate family; E340, personal care providers and personal assistants; E355, health professionals; E410, individual attitudes of immediate family members; E525, housing services, systems and policies; E580, health services, systems and policies.

## Discussion

The aim of the present study was to investigate the feasibility and effectiveness of the Moonwalker exoskeleton, a reciprocating aid system supporting upright standing and locomotion under gravitational load. Physically disabled individuals, such as children with CP, are often exposed to physical and interpersonal barriers, which decrease self-esteem and encourage learned helplessness (54). Gait impairments in children with CP may further exacerbate functional limitation and participation restriction (55), all of which are interconnected with the components of the International Classification of Functioning, Disability and Health (ICF) and the pediatric International Classification of Functioning, Disability and Health-Children and Youth (ICF-CY). Developed by the World Health Organization to establish a common language for understanding and investigating health and health-related states, the ICF and ICF-CY have gained increasing use in rehabilitation science as outcome measures (56–59).

Regular walking exercise generally enhances bone density, muscle strength, trunk and lower limb control, overall fitness, and coordination of trunk and leg motions (60, 61). In children with severe CP (most of them with GMFCS IV-V, Table 1) who cannot use a conventional walker, the Moonwalker may represent an indispensable training tool. In our study, the outcome measures included measurement of gait efficiency using the 10 Meter Walk Test and the levels of sitting postural stability in a subset of children (Table 2). Even though the device did not adversely affect children's developmental milestones, such as autonomous trunk control and unsupported walking, most children were able to use the Moonwalker both in the rehabilitation centre and in a domestic environment. They even improved endurance by traveling ten meters in less time or by increasing the distance walked in one minute following training (Table 2). Some of them achieved rather impressive results (e.g., child 15 failed to walk at T0 in the exoskeleton while reached 0.47 m/s at T2, some children reached a speed of more than 0.6 m/s at T2, Table 2), which is likely close to optimal and/or quite efficient for interaction with environment and mobility, given the constraints of the passive walking exoskeleton and the children's size. Training in the clinic improved the performance (T1) but training at home further increase the walking speed (T2, Figure 3).

The self-selected walking speed normally depends on the age of children due to body growth (62), although we did not find significant correlations between the achieved walking speed and the child' age, likely due to multiple factors affecting walking inability. While the dimensionless Froude number is typically used to compare the natural walking speed of children of different heights (63), walking speed optimisation (64) may be limited by the limitations imposed by the individual limb segment characteristics influenced by the endorsed exoskeleton. Examining the optimal walking speed while taking biomechanical constraints into account would be interesting, but interpreting altered biomechanics, muscle forces, and sensory inputs is challenging and necessitates a dynamic model of the human exoskeleton system as well as neuromechanical simulations with integrated sensory commands (65). Furthermore, important individual variations in exoskeleton stepping adaptation may exist. For instance, we noticed a variable involvement of the upper trunk, head and arm movements in different children (Figure 2). However, a registration of kinematic and kinetic parameters as well as muscle activity pattern would be necessary to assess particular modifications in the spinal locomotor output in order to better understand the neural control strategy when walking in the exoskeleton (41). Nevertheless, for our children's age range (2–8 years), the good attained speed was around 0.5 m/s: after training, 5 children remained with poor performance (<0.2 m/s), while the performance of the majority of them clustered around 0.3–0.8 m/s (Figure 3). It is also worth noting that the Moonwalker exoskeleton's achieved walking speed (∼0.5–0.7 m/s, Figure 3) was greater than the slow overground walking speed (∼0.1–0.3 m/s) that is usually employed with a powered exoskeleton (due to safety and stability issues) in clinical settings for children's gait assistance and rehabilitation (37, 41, 66). In sum, the 10-m walk test is a useful test to assess walking in the exoskeleton and the achieved walking speed may represent an important performance indicator of efficient interaction with environment and mobility when using the Moonwalker exoskeleton.

This study also demonstrated the compatibility and beneficial effects of the Moonwalker, as indicated by the results of the ICF-CY assessment in a subset of children (Table 3), consistent with previous studies that performed training of children with severe CP at home (45). It can indicate the success or failure of integrating an aid into the life of the child and their family and is crucial for assessing prescriptive adequacy (45, 47, 67), particularly when the functional profile was negatively influenced by environmental factors that shaped the training process. Kuenzle and Brunner (45) prioritized distance walked per day though they did not measure the maximal achieved performance (Figure 3) or LSS assessments. Other similar devices with a wheeled walker frame have been used for walking training in children with CP, but they either used motorized leg movements or conducted the tests only in a clinical setting (39, 40, 42–44, 46). With the help of the Moonwalker exoskeleton, children with significant posture and gait impairments can walk every day (two of our children with severe CP also used the Moonwalker to get to school), increasing their level of independence. They can engage in social activities and gain more freedom if they are able to walk with little to no help. Several positive environmental factors were noted and parents' expressed satisfaction (Table 3). In light of improvements in gait performance (Table 2) and favourable evaluations from end-users (children, parents, and medical personnel; Tables 3) the Moonwalker may eventually be used as a training aid for children with severe cerebral palsy at home and in the long run.

### Limitations of the study

This study has some limitations. First, the small sample size and heterogeneity of the participants limit the generalizability of the findings. Some evaluations were conducted only on a subset of participants (*n* = 7, Table 3), which limits the scope of the parental satisfaction and usability data. Nevertheless, even though we did not explicitly evaluate the feedback for all participants, all children completed the long-term training protocol (except for child 10, Table 2) based on the initial favourable considerations of physical and social conditions, and positive family support from the beginning and throughout the training sessions and at-home activities, promoting increasing independence and social interactions. Second, the study was not designed with groups undergoing only conventional therapy or only Moonwalker training. Each child in the sample continued their existing rehabilitative therapies throughout the study. Nevertheless, all of the children were receiving conventional therapy for a few months or years prior to being admitted to this study. Therefore, the baseline values at T0 reflect the benefits of ongoing conventional rehabilitation, while the final outcomes should likely be interpreted as the effects of the Moonwalker training. One should also take into account that we examined children with severe CP, who could not stand alone and use conventional walker. Finally, we started training at the age of >2 years (Table 1), in part because of certain limitations, including minimum body height requirements (the Moonwalker requires a minimum height of 80 cm) and the early identification of potential candidates who could benefit from the device. Given critical developmental windows, it would be interesting to begin locomotor therapy even sooner (<2 years old), and clinicians working with children with CP should take into account the possible effects of starting training earlier (19, 33–35, 44).

## Conclusions

Mobility is an appealing aim since it is important for people, especially for children who are not ambulatory. Future studies on gait therapy may concentrate on developing technology frameworks. In addition to teaching gait patterns, these technologies need to help children navigate their environment and provide them engaging, cognitive experiences. The results of the 10-m walking test, employed in this study, seem to represent important performance indicators to assess the potential of the Moonwalker to enhance upright standing and locomotion in children with severe CP, thereby fostering greater autonomy and participation in daily life activities. Based on the results (Figure 3), we suggest that the walking speed of ∼0.5–0.7 m/s (depending on age) can be considered as a desirable goal for monitoring improvements of walking mobility and endurance following training in this type of exoskeleton in children with severe CP. Also, such speeds enable effective movement and participation in daily activities, promoting increasing independence and social interactions. These findings advocate for larger controlled intervention studies to further explore the long-term efficacy of robotic assistive devices in improving gait and functional outcomes in children with CP in a domestic environment.

## Data Availability

The original contributions presented in the study are included in the article/Supplementary Material, further inquiries can be directed to the corresponding author.

## References

[1] Bax M Goldstein M Rosenbaum P Leviton A Paneth N Dan B Proposed definition and classification of cerebral palsy, April 2005. Dev Med Child Neurol. (2005) 47:571–6. 10.1017/S001216220500112X 16108461

[2] Poon DMY Hui-Chan CWY. Hyperactive stretch reflexes, co-contraction, and muscle weakness in children with cerebral palsy. Dev Med Child Neurol. (2009) 51:128–35. 10.1111/j.1469-8749.2008.03122.x 19018843

[3] Christensen D Van Naarden Braun K Doernberg NS Maenner MJ Arneson CL Durkin MS Prevalence of cerebral palsy, co-occurring autism spectrum disorders, and motor functioning—autism and developmental disabilities monitoring network, USA, 2008. Dev Med Child Neurol. (2014) 56:59–65. 10.1111/dmcn.12268 24117446 PMC4351771

[4] Rosenbaum P Eliasson A-C Hidecker MJC Palisano RJ. Classification in childhood disability: focusing on function in the 21st century. J Child Neurol. (2014) 29:1036–45. 10.1177/0883073814533008 24810083

[5] Sellier E Platt MJ Andersen GL Krägeloh-Mann I De La Cruz J Cans C Decreasing prevalence in cerebral palsy: a multi-site European population-based study, 1980 to 2003. Dev Med Child Neurol. (2016) 58:85–92. 10.1111/dmcn.12865 26330098

[6] Hutton JL Pharoah PO. Effects of cognitive, motor, and sensory disabilities on survival in cerebral palsy. Arch Dis Child. (2002) 86:84–9. 10.1136/adc.86.2.84 11827899 PMC1761069

[7] Willoughby KL Dodd KJ Shields N. A systematic review of the effectiveness of treadmill training for children with cerebral palsy. Disabil Rehabil. (2009) 31:1971–9. 10.3109/09638280902874204 19874075

[8] Smania N Bonetti P Gandolfi M Cosentino A Waldner A Hesse S Improved gait after repetitive locomotor training in children with cerebral palsy. Am J Phys Med Rehabil. (2011) 90:137–49. 10.1097/PHM.0b013e318201741e 21217461

[9] Degelean M De Borre L Salvia P Pelc K Kerckhofs E De Meirleir L Effect of ankle-foot orthoses on trunk sway and lower limb intersegmental coordination in children with bilateral cerebral palsy. J Pediatr Rehabil Med. (2012) 5:171–9. 10.3233/PRM-2012-0209 23023249

[10] Graham HK Rosenbaum P Paneth N Dan B Lin J-P Damiano DL Cerebral palsy. Nat Rev Dis Primers. (2016) 2:15082. 10.1038/nrdp.2015.82 27188686 PMC9619297

[11] Whittingham K Fahey M Rawicki B Boyd R. The relationship between motor abilities and early social development in a preschool cohort of children with cerebral palsy. Res Dev Disabil. (2010) 31:1346–51. 10.1016/j.ridd.2010.07.006 20674264

[12] Anderson DI Campos JJ Witherington DC Dahl A Rivera M He M The role of locomotion in psychological development. Front Psychol. (2013) 4:440. 10.3389/fpsyg.2013.00440 23888146 PMC3719016

[13] Palisano RJ Tieman BL Walter SD Bartlett DJ Rosenbaum PL Russell D Effect of environmental setting on mobility methods of children with cerebral palsy. Dev Med Child Neurol. (2003) 45:113–20. 10.1111/j.1469-8749.2003.tb00914.x 12578237

[14] McEwen IR. Assistive positioning as a control parameter of social-communicative interactions between students with profound multiple disabilities and classroom staff. Phys Ther. (1992) 72:634–44. discussion 644-647. 10.1093/ptj/72.9.634 1508971

[15] Lancioni GE Singh NN O’Reilly MF Sigafoos J Didden R Manfredi F Fostering locomotor behavior of children with developmental disabilities: an overview of studies using treadmills and walkers with microswitches. Res Dev Disabil. (2009) 30:308–22. 10.1016/j.ridd.2008.05.002 18573637

[16] Cappellini G Sylos-Labini F MacLellan MJ Sacco A Morelli D Lacquaniti F Backward walking highlights gait asymmetries in children with cerebral palsy. J Neurophysiol. (2018) 119:1153–65. 10.1152/jn.00679.2017 29357466

[17] Cappellini G Sylos-Labini F MacLellan MJ Assenza C Libernini L Morelli D Locomotor patterns during obstacle avoidance in children with cerebral palsy. J Neurophysiol. (2020) 124(2):574–90. 10.1152/jn.00163.2020 32667246

[18] Cappellini G Sylos-Labini F Avaltroni P Dewolf AH Assenza C Morelli D Comparison of the forward and sideways locomotor patterns in children with cerebral palsy. Sci Rep. (2023) 13:7286. 10.1038/s41598-023-34369-4 37142631 PMC10160037

[19] Cappellini G Sylos-Labini F Dewolf AH Solopova IA Morelli D Lacquaniti F Maturation of the locomotor circuitry in children with cerebral palsy. Front Bioeng Biotechnol. (2020) 8:998. 10.3389/fbioe.2020.00998 32974319 PMC7462003

[20] Kerr C McDowell BC Parkes J Stevenson M Cosgrove AP. Age-related changes in energy efficiency of gait, activity, and participation in children with cerebral palsy. Dev Med Child Neurol. (2011) 53:61–7. 10.1111/j.1469-8749.2010.03795.x 20875041

[21] Day JA Fox EJ Lowe J Swales HB Behrman AL. Locomotor training with partial body weight support on a treadmill in a nonambulatory child with spastic tetraplegic cerebral palsy: a case report. Pediatr Phys Ther. (2004) 16:106–13. 10.1097/01.PEP.0000127569.83372.C8 17057535

[22] Meyer-Heim A Ammann-Reiffer C Schmartz A Schäfer J Sennhauser FH Heinen F Improvement of walking abilities after robotic-assisted locomotion training in children with cerebral palsy. Arch Dis Child. (2009) 94:615–20. 10.1136/adc.2008.145458 19208675

[23] Azizi S Marzbani H Raminfard S Birgani PM Rasooli AH Mirbagheri MM. The impact of an anti-gravity treadmill (AlterG) training on walking capacity and corticospinal tract structure in children with cerebral palsy. Annu Int Conf IEEE Eng Med Biol Soc. (2017) 2017:1150–3. 10.1109/EMBC.2017.8037033 29060079

[24] Wallard L Dietrich G Kerlirzin Y Bredin J. Robotic-assisted gait training improves walking abilities in diplegic children with cerebral palsy. Eur J Paediatr Neurol. (2017) 21:557–64. 10.1016/j.ejpn.2017.01.012 28188024

[25] Aycardi LF Cifuentes CA Múnera M Bayón C Ramírez O Lerma S Evaluation of biomechanical gait parameters of patients with cerebral palsy at three different levels of gait assistance using the CPWalker. J Neuroeng Rehabil. (2019) 16:15. 10.1186/s12984-019-0485-0 30691493 PMC6350321

[26] Solopova IA Sukhotina IA Zhvansky DS Ikoeva GA Vissarionov SV Baindurashvili AG Effects of spinal cord stimulation on motor functions in children with cerebral palsy. Neurosci Lett. (2017) 639:192–8. 10.1016/j.neulet.2017.01.003 28063935

[27] Ivanenko Y Shapkova EY Petrova DA Kleeva DF Lebedev MA. Exoskeleton gait training with spinal cord neuromodulation. Front Hum Neurosci. (2023) 17:1194702. 10.3389/fnhum.2023.1194702 37250689 PMC10213721

[28] Willerslev-Olsen M Petersen TH Farmer SF Nielsen JB. Gait training facilitates central drive to ankle dorsiflexors in children with cerebral palsy. Brain. (2015) 138:589–603. 10.1093/brain/awu399 25623137 PMC4408439

[29] Lerner ZF Damiano DL Bulea TC. The effects of exoskeleton assisted knee extension on lower-extremity gait kinematics, kinetics, and muscle activity in children with cerebral palsy. Sci Rep. (2017) 7:13512. 10.1038/s41598-017-13554-2 29044202 PMC5647342

[30] Hunt M Everaert L Brown M Muraru L Hatzidimitriadou E Desloovere K. Effectiveness of robotic exoskeletons for improving gait in children with cerebral palsy: a systematic review. Gait Posture. (2022) 98:343–54. 10.1016/j.gaitpost.2022.09.082 36306544

[31] Forma V Anderson DI Provasi J Soyez E Martial M Huet V What does prone skateboarding in the newborn tell us about the ontogeny of human locomotion? Child Dev. (2019) 90:1286–302. 10.1111/cdev.13251 31267516

[32] Kolobe THA Fagg AH. Robot reinforcement and error-based movement learning in infants with and without cerebral palsy. Phys Ther. (2019) 99:677–88. 10.1093/ptj/pzz043 31155667 PMC6545273

[33] Yang JF Livingstone D Brunton K Kim D Lopetinsky B Roy F Training to enhance walking in children with cerebral palsy: are we missing the window of opportunity? Semin Pediatr Neurol. (2013) 20:106–15. 10.1016/j.spen.2013.06.011 23948685

[34] Friel KM Williams PTJA Serradj N Chakrabarty S Martin JH. Activity-Based therapies for repair of the corticospinal system injured during development. Front Neurol. (2014) 5:229. 10.3389/fneur.2014.00229 25505443 PMC4241838

[35] Hadders-Algra M. Early diagnosis and early intervention in cerebral palsy. Front Neurol. (2014) 5:185. 10.3389/fneur.2014.00185 25309506 PMC4173665

[36] De Luca R Bonanno M Settimo C Muratore R Calabrò RS. Improvement of gait after robotic-assisted training in children with cerebral palsy: are we heading in the right direction? Med Sci (Basel). (2022) 10:59. 10.3390/medsci10040059 36278529 PMC9624362

[37] Kuroda MM Iwasaki N Mutsuzaki H Yoshikawa K Takahashi K Nakayama T Benefits of a wearable cyborg HAL (hybrid assistive limb) in patients with childhood-onset motor disabilities: a 1-year follow-up study. Pediatr Rep. (2023) 15:215–26. 10.3390/pediatric15010017 36976724 PMC10057157

[38] Choi JY Kim SK Hong J Park H Yang S-S Park D Overground gait training with a wearable robot in children with cerebral palsy: a randomized clinical trial. JAMA Netw Open. (2024) 7:e2422625. 10.1001/jamanetworkopen.2024.22625 39037815 PMC11265136

[39] Delgado E Cumplido C Ramos J Garcés E Puyuelo G Plaza A ATLAS2030 Pediatric gait exoskeleton: changes on range of motion, strength and spasticity in children with cerebral palsy. A case series study. Front Pediatr. (2021) 9:753226. 10.3389/fped.2021.753226 34900862 PMC8652111

[40] Castro P Martí M Oliván-Blázquez B Boñar N García V Gascón-Santos S Benefits of robotic gait assistance with ATLAS 2030 in children with cerebral palsy. Front Pediatr. (2024) 12:1398044. 10.3389/fped.2024.1398044 39135857 PMC11318455

[41] Villani M Avaltroni P Scordo G Rubeca D Berger DJ Kreynin P Evaluation of EMG patterns in children during assisted walking in the exoskeleton. Front. Neurosci. (2024) 18. 10.3389/fnins.2024.1461323 39513047 PMC11541598

[42] McCormick AM Alazem H Zaidi S Barrowman NJ Ward LM McMillan HJ A randomized, cross-over trial comparing the effect of innovative robotic gait training and functional clinical therapy in children with cerebral palsy; a protocol to test feasibility. Contemp Clin Trials. (2023) 127:107086. 10.1016/j.cct.2023.107086 36669727

[43] Bayón C Martín-Lorenzo T Moral-Saiz B Ramírez Ó Pérez-Somarriba Á Lerma-Lara S A robot-based gait training therapy for pediatric population with cerebral palsy: goal setting, proposal and preliminary clinical implementation. J Neuroeng Rehabil. (2018) 15:69. 10.1186/s12984-018-0412-9 30053857 PMC6063005

[44] Wright FV Jutai JW. Evaluation of the longer-term use of the David Hart Walker orthosis by children with cerebral palsy: a 3-year prospective evaluation. Disabil Rehabil Assist Technol. (2006) 1:155–66. 10.1080/17483100600627382 19260183

[45] Kuenzle C Brunner R. The effects of the norsk funktion-walking orthosis on the walking ability of children with cerebral palsy and severe gait impairment. JPO: J Prosthet Orthot. (2009) 21:138. 10.1097/JPO.0b013e3181b173ec

[46] Smania N Gandolfi M Marconi V Calanca A Geroin C Piazza S Applicability of a new robotic walking aid in a patient with cerebral palsy. Case report. Eur J Phys Rehabil Med. (2012) 48:147–53. 22543558

[47] Henderson S Skelton H Rosenbaum P. Assistive devices for children with functional impairments: impact on child and caregiver function. Dev Med Child Neurol. (2008) 50:89–98. 10.1111/j.1469-8749.2007.02021.x 18177410

[48] Paleg GS Williams SA Livingstone RW. Supported standing and supported stepping devices for children with non-ambulant cerebral palsy: an interdependence and F-words focus. Int J Environ Res Public Health. (2024) 21:669. 10.3390/ijerph21060669 38928915 PMC11203597

[49] Chrysagis N Skordilis EK Koutsouki D. Validity and clinical utility of functional assessments in children with cerebral palsy. Arch Phys Med Rehabil. (2014) 95:369–74. 10.1016/j.apmr.2013.10.025 24239880

[50] Chung J Evans J Lee C Lee J Rabbani Y Roxborough L Effectiveness of adaptive seating on sitting posture and postural control in children with cerebral palsy. Pediatr Phys Ther. (2008) 20:303–17. 10.1097/PEP.0b013e31818b7bdd 19011521

[51] Field DA Roxborough LA. Responsiveness of the seated postural control measure and the level of sitting scale in children with neuromotor disorders. Disabil Rehabil Assist Technol. (2011) 6:473–82. 10.3109/17483107.2010.532285 21110727

[52] Demers L Weiss-Lambrou R Ska B. Item analysis of the Quebec user evaluation of satisfaction with assistive technology (QUEST). Assist Technol. (2000) 12:96–105. 10.1080/10400435.2000.10132015 11508406

[53] Stucki G Kostanjsek N Ustün B Cieza A. ICF-based classification and measurement of functioning. Eur J Phys Rehabil Med. (2008) 44:315–28. 18762741

[54] Avillion AE. Barrier perception and its influence on self-esteem. Rehabil Nurs. (1986) 11:11–4. 10.1002/j.2048-7940.1986.tb00514.x 2944195

[55] Lee J-W Chung E Lee B-H. A comparison of functioning, activity, and participation in school-aged children with cerebral palsy using the manual ability classification system. J Phys Ther Sci. (2015) 27:243–6. 10.1589/jpts.27.243 25642083 PMC4305573

[56] Schiariti V Klassen AF Cieza A Sauve K O’Donnell M Armstrong R Comparing contents of outcome measures in cerebral palsy using the international classification of functioning (ICF-CY): a systematic review. Eur J Paediatr Neurol. (2014) 18:1–12. 10.1016/j.ejpn.2013.08.001 24051208

[57] Schroeder AS Homburg M Warken B Auffermann H Koerte I Berweck S Prospective controlled cohort study to evaluate changes of function, activity and participation in patients with bilateral spastic cerebral palsy after robot-enhanced repetitive treadmill therapy. Eur J Paediatr Neurol. (2014) 18:502–10. 10.1016/j.ejpn.2014.04.012 24821475

[58] Lexell J Brogårdh C. The use of ICF in the neurorehabilitation process. NeuroRehabilitation. (2015) 36:5–9. 10.3233/NRE-141184 25547759

[59] Hsieh Y-L Yang C-C Sun S-H Chan S-Y Wang T-H Luo H-J. Effects of hippotherapy on body functions, activities and participation in children with cerebral palsy based on ICF-CY assessments. Disabil Rehabil. (2017) 39:1703–13. 10.1080/09638288.2016.1207108 27440177

[60] Schindl MR Forstner C Kern H Hesse S. Treadmill training with partial body weight support in nonambulatory patients with cerebral palsy. Arch Phys Med Rehabil. (2000) 81:301–6. 10.1016/S0003-9993(00)90075-3 10724074

[61] Blundell SW Shepherd RB Dean CM Adams RD Cahill BM. Functional strength training in cerebral palsy: a pilot study of a group circuit training class for children aged 4–8 years. Clin Rehabil. (2003) 17:48–57. 10.1191/0269215503cr584oa 12617379

[62] Ivanenko YP Dominici N Cappellini G Dan B Cheron G Lacquaniti F. Development of pendulum mechanism and kinematic coordination from the first unsupported steps in toddlers. J Exp. Biol. (2004) 207:3797–810. 10.1242/jeb.01214 15371487

[63] Cavagna GA Franzetti P Fuchimoto T. The mechanics of walking in children. J Physiol (Lond). (1983) 343:323–39. 10.1113/jphysiol.1983.sp014895 6644619 PMC1193922

[64] Leurs F Ivanenko YP Bengoetxea A Cebolla A-M Dan B Lacquaniti F Optimal walking speed following changes in limb geometry. J Exp. Biol. (2011) 214:2276–82. 10.1242/jeb.054452 21653821

[65] Di Russo A Stanev D Sabnis A Danner SM Ausborn J Armand S Investigating the roles of reflexes and central pattern generators in the control and modulation of human locomotion using a physiologically plausible neuromechanical model. J Neural Eng. (2023) 20. 10.1088/1741-2552/acfdcc 37757805

[66] Karunakaran KK Ehrenberg N Cheng J Bentley K Nolan KJ. Kinetic gait changes after robotic exoskeleton training in adolescents and young adults with acquired brain injury. Appl Bionics Biomech. (2020) 2020:8845772. 10.1155/2020/8845772 33193810 PMC7641681

[67] Hocking C. Function or feelings: factors in abandonment of assistive devices. Technol Disabil. (1999) 11:3–11. 10.3233/TAD-1999-111-202

